# Self-harming behaviors and forensic system-related factors: an analysis of the Ontario review board database

**DOI:** 10.1186/s12888-023-05394-4

**Published:** 2023-12-06

**Authors:** Mark Mohan Kaggwa, Gary Andrew Chaimowitz, Bailea Erb, Sébastien Prat, Arianna Davids, Heather Moulden, Amara Robbins, John Bradford, Mini Mamak, Andrew Toyin Olagunju

**Affiliations:** 1 Forensic Psychiatry Program, St Joseph’s Healthcare Hamilton, 100 West 5th, Hamilton, ON L89 3K7 Canada; 2 Department of Psychiatry and Behavioral Sciences, McMaster University, St Joseph’s Healthcare Hamilton, 100 West 5th, Hamilton, ON L89 3K7 Canada; 3 Mbarara University of Science and Technology, Mbarara, Uganda; 4 Division of Forensic Psychiatry, University of Ottawa, Ottawa, ON Canada; 5 Discipline of Psychiatry, University of Adelaide, Adelaide, SA 5000 Australia

**Keywords:** Forensic, Ontario Review Board, Self-harm, Suicidal behaviors, Unfit to stand Trial

## Abstract

**Background:**

In Canada, ensuring public safety, and the safety and well-being of accused individuals under the jurisdiction of the provincial review board are very important. While previous studies have reported a significant risk of self-harming behaviors (non-suicidal self-injury and suicide attempt) in forensic psychiatric settings, no large population study has assessed any relationship between forensic system-related factors and self-harming behaviors. A better understanding of these factors can help clinicians implement protective measures to mitigate self-harming behaviors or actions.

**Methods:**

Using the Ontario Review Board (ORB) database covering 2014–2015 period (n = 1211, mean age = 42.5 ± 13.37 years, males = 86.1%), we analyzed the prevalence and factors associated with self-harming behaviors, emphasizing the characterization of the forensic system-related factors (ORB status, legal status, type of offense, previous criminal history, and victim relationship). The relationships between the forensic system-related factors and self-harming behaviors were explored using five separate logistic regression models, controlling for clinical and sociodemographic characteristics.

**Results:**

Approximately 4% of the individuals in the forensic system over the study period engaged in self-harming behaviors Among the studied patients, individuals determined to be unfit to stand trial and inpatients were significantly more likely to have self-harming behaviors. There was no significant relationship between the type of offence, victim relationship, and previous criminal history with self-harming behavior.

**Conclusion:**

Forensic psychiatry inpatients should have close observation, screening, monitoring, and individual tailored management strategies for self-harming behaviors. The findings of this study indicate that forensic system-related factors, especially those that pertain to the status of individuals in the forensic system (i.e., unfit to stand trial and being an inpatient) are more responsible for self-harming behaviors among forensic patients in Ontario.

## Introduction

In Canada, the forensic psychiatric system represents an important component of the public mental health system, that conducts special assessments and provides care to legally involved individuals with mental illness who are found unfit to stand trial (UST) or not criminally responsible due to mental illness (NCR) [1–3]. Both UST and NCR are status in forensic psychiatric system that are determined by a court. In the determination of both UST and NCR, the court often incorporates expert opinion of mental health professionals and findings from psycho-legal assessments [2, 3]. While the psycho-legal assessments to ascertain UST pertains to the individual’s current mental status at any stage of the trial proceeding before a verdict is rendered, NCR assessment is focused on the mental state of an individual at the time of the alleged offence [1–4]. Diagnostically, UST and NCR are similar by virtue of the significant contributions of mental disorders to their determination, albeit more individuals with acquired brain injuries and intellectual disorders are often found UST [4]. Some of the active symptoms of severe mental health conditions experienced by individuals found UST or NCR in addition to the offences committed may lead to some of them being found a risk to public safety [1, 3].

While public safety is a paramount goal of forensic mental services, the safety and well-being of individuals within the forensic system are also sacrosanct. Incidents of life-threatening behaviors or deaths within the forensic system can have serious ramifications on co-patients, families, staff members, and the program, and may trigger an audit or special scrutiny of the affected program to allow prevention of future incidents [5, 6].

Patients in the forensic psychiatry system often require substantial effort and specialized skill sets in risk management, as well as health care management to address complex mental health phenomena and comorbid psychosocial problems (e.g., antisocial behaviors, early adverse experiences, and substance use) that are prevalent among the population [7]. While individuals in the forensic psychiatric system have been linked to a significant degree of risk for suicidal, self-harm, and harmful-risky behaviors [7, 8], only a few studies have explored these phenomena among forensic populations. By way of definition, self-harm is the act of hurting yourself on purpose, including intentional self-poisoning or self-injury irrespective of the apparent purpose of the act [9, 10].

Available studies among individuals in forensic psychiatry system have reported alarming rates of self-harming behaviors, ranging between 36 and 68.4% [7, 8, 11–14]. A recent study from Greenland reported that over a third of forensic patients attempted suicide in their lifetime [8]. Several types of self-harming behaviors reported in previous studies in forensic settings (e.g., hanging, self-poisoning, cutting, self-strangulation, choking or swallowing objects, jumping from heights, traffic-related attempts, recklessly getting an infection, running out on an iced lake, starting a fire, drug overdose and injecting air into their blood among others) have resulted in deaths, and major or permanent life-changing consequences [7, 8, 13, 15].

The high prevalence of self-harming behaviors and lethality of the self-harming methods used among forensic patients warrant early identification, appropriate interventions and monitoring, as approximately 10% of attempters die by suicide within ten years [16]. Several programs have been designed or adapted to mitigate the risk of self-harming behaviors in forensic settings, such as environmental modifications (e.g., minimizing fixtures, avoiding ligature points, and reducing breakable and pointed objects.), routine monitoring, and use of pharmacological interventions [17]. A recent systematic review and meta-analysis indicated that these interventions resulted in significant reduction in self-harming behaviors among individuals in a correctional facility, although prevention of suicide and self-harm still presents important challenges [18].

In addition to the above mentioned interventions, identifiable factors (such as older age, younger age at the onset of mental health-related problems, index offense, severe psychopathology, higher levels of depression and anxiety, adverse childhood events, emotional abuse among females, substance use, and a criminal conviction) can be used in the prediction of self-harming behaviors and inform preventive interventions, [8, 12, 19]. In relation to a similar population (i.e., individuals in prison), a systematic review and meta-analysis published in the Lancet also reported suicide-related antecedents, sociodemographic, criminological factors, and current psychiatry diagnosis (depression and borderline personality disorder) to be associated with self-harm [20]. While acknowledging the importance of previous studies to improving the understanding of the predictors of self-harming behaviors, the existing literature is limited due to insufficient depth, small samples, and a failure to explore potentially modifiable factors within forensic facilities or systems. Also, the findings from previous studies among forensic patients regarding clinical and sociodemographic risk factors associated with self-harming behaviors are similar to other settings or mental health patient populations. However, factors regarding the risks for self-harming associated with the forensic system have not been explored. The current study is therefore aimed to determine the prevalence of self-harming behaviors and the associated forensic system related-factors (including ORB status, legal status, type of offense, previous criminal history, and victim relationship) using a large database of forensic inpatients and outpatients in Ontario, Canada – Ontario Review Board (ORB) database [3]. The forensic system-related factors are closely linked to the criminological and risk management aspects of forensic psychiatric services and explore the status of the patients while in the forensic system.

## Methods

### Study population

Using the ORB database for the period covering 2014 and 2015 [3], we analyzed information related to self-harming behaviors (non-suicidal self-injury and suicide attempt). We excluded individuals with missing information on self-harm (n = 29). A total of 1211 eligible patients were included in this study for final analysis. The ORB database and the characteristics of individuals in the database have been described in a previous study [3]. Briefly, the ORB database was created using information extracted on predetermined variables from the reports submitted to the ORB from all forensic psychiatric programs (n = 12) in the province of Ontario during 2014 and 2015. These ORB reports are detailed summaries prepared annually by the hospital-based team for all forensic patients based on clinical observations, assessments, and progress through rehabilitation programs.

Upon the finding of UST or NCR and detention in a forensic program in Ontario by a court, the ORB assumes jurisdictions over individuals detained in the forensic system, providing oversight role and make or review dispositions (which broadly include detention, conditional discharge, and absolute discharge order). The disposition also includes guidance on treatment, management and the latitude of privileges approved for individual forensic patients to promote their recovery, risk management, rehabilitation, and safe re-integration into the community. A multi-disciplinary team of mental health professionals work with forensic patients, conducting assessments using validated tools, developing programs or care plans to manage risk, enhancing recovery, and facilitating community re-integration. Annually, a report is prepared by the team on individual forensic patients as evidence for deliberation during ORB hearings. The reports provide one of the best snapshots on all individuals detained in the Ontario forensic psychiatric system and are submitted as evidence for patients’ progress, major incidents, assessments conducted, care plans or transition within the forensic psychiatry system. The reports contribute majorly to the evidence used by the ORB to inform decisions on new dispositions and support for the care of individuals in the forensic psychiatric system in the province of Ontario [3].

### Identifying self-harming behaviors

Information on self-harming behaviour was completed based on a variable that captures self-harming behaviour during the reporting year under the ORB system. Patients under the ORB system are on active monitoring, whether inpatients or outpatients, and incidents of self-harming behaviors are captured in close proximity to their happening. These events are documented in the ORB report as part of the summary evidence for the reporting year. The variable for self-harming behaviors was operationalised in this study with a *Yes or No response*. A *yes* indicated presence of self-harming behaviour (i.e., intentional self-harm or suicide attempts) during the 12-month reporting period.

### Covariates

All covariates were chosen based on the information available from the database and potential confounder of the relationship between self-harming behaviour and forensic system-related characteristics. Demographic variables (age and gender) and clinical characteristics, including lifetime history of substance use (for addictive substances including alcohol and marijuana), previous hospitalization in psychiatric institutions, prior self-harm behavior before the reporting year, current primary mental health diagnosis made by the patients’ psychiatrist based on Diagnostic and Statistical Manual of Mental Disorders, and presence of a current comorbid psychiatric diagnosis were also included.

The forensic system-related characteristics included: (i) review board status (UST or NCR), (ii) legal status (inpatient secure [admitted on units with medium to high security with minimal levels of freedom exercised by patient], inpatient general [admitted to a minimum level of restriction unit], or outpatient), (iii) previous involvement with the criminal justice system, (iv) relationship with the victim of the index offence, and (v) type of index offence committed (among the offences the first subjectively considered offence was categorised).

The type of initial index offense was classified into three: (i) violent (murder -including attempted murder; assault - including assault causing bodily harm and aggravated assault; robbery - bank, store, and purse snatching; abduction - including attempted abduction; threatening with a weapon; and verbal threats), (ii) non-violent/general offences (criminal harassment; arson and fire setting; theft - includes car theft and possession of stolen property; mischief to public or private property; break and enter and commit an indictable offence [burglary]; breaking and entering with intent to comment an offence; fraud [extortion, embezzlement, forged check, impersonation]; possession of prohibited or restricted weapon; procuring a person for, or living on the avails of prostitution; trafficking in Narcotics; driving-related offences [dangerous driving, impaired driving, driving while ability impaired]; obstructing peace officer, obstruct justice, breach probation, failure to comply, resist arrest, and escape custody), and sexual (sexual assault, sexual interference, indecent assault, and indecent act or exposure).

### Ethical approval and consent

The study was conducted under the Declaration of Helsinki. The present study was approved by Hamilton Integrated Research Ethics Board (HiREB) reference: #15,564. The need for informed consent was waived by the ethics committee/institutional review board of Hamilton, Ontario institutions i.e., the Hamilton Integrated Research Ethics Board (HiREB).

### Statistical analyses

Data were analyzed using STATA version 17.0. Descriptive statistics of the study variables on individuals who were reported to have experienced self-harming behaviors compared with those without were presented in term of percentages and frequencies for categorical variables and mean (± standard deviation) for age. Likewise, deletion method was applied to address missing data [21, 22]. Statistical differences between variables were compared using the chi-square test and t-test. Five separate logistic regression models were used to determine the forensic system-related factors (including ORB status, legal status, type of offense, previous criminal history, and victim relationship) that are independently associated with self-harming behaviors. First, the univariate logistic regression was used to determine the relationship strength. This was followed by a second regression analysis to control for clinical and demographic factors and determine the forensic system-related factors associated with self-harming behaviors. Variance Inflation Factor (< 2) was used to test for collinearity. A p-value < 0.05 was set as statistical significance with a 95% confidence interval.

## Results

### Comparison of individuals with and without self-harming behaviours

A total of 1211 study patients’ records (mean age 42.5 [13.37] and 86.1% were males) were included in the study for the reporting period. A total of 43 patients (3.6%) had self-harming behaviors within the reporting period. Self-harming behaviors were observed more among those with a previous history of self-harm (16.2%) than those without (16.2% vs. 1.2%, χ^2^ = 101.00, *p*-value < 0.001). Comorbidity of psychiatry illnesses were also more common among individuals with self-harming behaviors than those without (4.1% vs. 2.0%, χ^2^ = 58.33, *p*-value < 0.001). There was also a statistically significant difference between the diagnosis of the individuals with and without self-harming behaviours (χ2 = 58.33, p-value < 0.001). The most common diagnosis among individuals with self-harming behaviors was neurodevelopmental disorders (15.2%), followed by personality disorders (9.4%). Regarding forensic system-related factors, there were statistical differences between review board status, legal status, and relationship to the victim. (Table 1).

**Table 1:** Comparison of clinical and sociodemographic characteristics of individuals with and without self-harming behaviours

Variable		Self-harming behaviors		
		No n (%) 1168 (96.4%)	Yes n (%) 43 (3.6%)	t/χ^2^ (*p*-value)
**Demographic characteristics**				
Age (mean, standard deviation)		42.7 (13.4)	38.8 (12.2)	1.88 (0.061)
Gender	Male	1003 (96.4)	37 (3.6)	0.11 (0.946)
	Female	162 (96.4)	6 (3.6)	
	Transgender	3 (100)	0	
**Clinical characteristics**				
History of substance abuse	No	325 (95.3)	16 (4.7)	2.15 (0.142)
	Yes	817 (97.0)	25 (3.0)	
Previous hospitalization	No	181 (96.8)	6 (3.2)	0.03 (0.861)
	Yes	976 (96.5)	35 (3.5)	
History of self-harming	No	984 (98.8)	12 (1.20)	101.00 (< 0.001)
	Yes	150 (83.8)	29 (16.2)	
Primary psychiatry diagnosis	Psychosis	958 (97.1)	29 (2.94)	58.33 (< 0.001)
	Mood disorders	79 (97.5)	2 (2.47)	
	Neurodevelopmental disorder	28 (84.8)	5 (15.2)	
	Personality disorder	48 (90.6)	5 (9.4)	
	Others	55 (96.5)	2 (3.5)	
Comorbid psychiatry illness	No	295 (98.0)	6 (2.0)	19.68 (0.001)
	Yes	873 (95.9)	37 (4.1)	
**Forensic-related factors**				
Review board status	Unfit to stand trial (UST)	94 (92.2)	8 (7.8)	5.99 (0.014)
	Not criminally responsible (NCR)	1074 (96.8)	35 (3.2)	
Legal status	Inpatient Secure	268 (91.2)	26 (8.80)	38.04 (< 0.001)
	Inpatient General	323 (96.1)	13 (3.90)	
	Outpatient	577 (99.3)	4 (0.7)	
Previous involvement with the criminal system	No	604 (96.8)	20 (3.2)	0.45 (0.503)
	Yes	564 (96.1)	23 (3.9)	
Type of offense	Violent	810 (95.9)	35 (4.1)	2.91 (0.233)
	Non-violent	242 (98.0)	5 (2.0)	
	Sexual	116 (97.5)	3 (2.5)	
Victim relationship	Stranger	317 (97.8)	7 (2.2)	3.89 (0.040)
	Known to patient	578 (95.2)	29 (4.8)	

### Factors that were independently associated with self-harming behaviors following regression analysis

At bivariate analysis, unfit to stand trial, review board status and being an inpatient (general or secure unit) were the forensic system-related factors significantly associated with increasing the likelihood of self-harming behaviors. After controlling for demographic and clinical factors, the same factors remained statistically significantly associated with higher odds for self-harming behaviors. (Table 2)

**Table 2:** Logistic regression analysis for factors associated with self-harming behaviors

Variable	Crude odds ratio (95% confidence interval)	*p-*values	Adjusted odds ratio (95% confidence interval)	*p*-value
**Review board status**				
Unfit to stand trial (UST)	2.61 (1.18–5.79)	**0.018**	3.00 (1.06–8.52)	**0.039**
Not criminally responsible (NCR)	1 (reference)		1 (reference)	
**Legal status**				
Inpatient Secure	13.99 (4.84–40.50)	**< 0.001**	12.95 (4.09–41.03)	**< 0.001**
Inpatient General	5.81 (1.88–17.95)	**0.002**	5.62 (1.70–18.59)	**0.005**
Outpatient	1 (reference)		1 (reference)	
**Previous involvement with the criminal system**				
No	1 (reference)		1 (reference)	
Yes	1.23 (0.67–2.26)	0.503	1.03 (0.49–2.16)	0.936
**Type of offense**				
Violent	2.09 (0.81–5.40)	0.127	2.23 (0.75–6.67)	0.151
Non-violent	1 (reference)		1 (reference)	
Sexual	1.25 (0.29–5.32)	0.761	1.10 (0.21–5.69)	0.907
**Victim relationship**				
Stranger	1 (reference)		1 (reference)	
Known to patient	2.27 (0.98–5.24)	0.055	1.98 (0.81–4.84)	0.136

All models were controlled for demographic (age and gender) and clinical characteristics (history of substance use, previous hospitalization, history of self-harm, diagnosis, and presence of a comorbid psychiatry diagnosis)

## Discussion

In the present study, approximately 4% of individuals under the ORB had engaged in self-harming behaviors over the reported period. This prevalence is lower than those reported in previous studies, and may be attributed to their small sample size (below 100 individuals) and exclusion of outpatients (i.e., patients in high secure facility in Sweden and minimum secure unit in UK) in the previous studies cited [7, 8, 11, 12]. In addition to the small sample sizes, the operational definitions of self-harming behaviors in previous studies cited varied from the current study which might have led to the differences in the prevalence rate. For example, while the present study examined the prevalence of self-harming over 12-month, some of the cited studies reported only suicide attempts in the past six months [7], another reported both self-harming behaviors and suicide attempts in the past six months [11], and others captured both self-harming behaviors since the age of 18 [12] or lifetime [8]. Capturing self-harming behaviors beyond one year can lead to a higher reported prevalence compared to the current study. Considering that risk management (e.g., risk of violence and self-harming behaviour) is one of the major role of the forensic psychiatric system [24], it is rational to assume that the burden or prevalence would change over time due to treatment and other management modalities. Consequently, capturing recent incidents of self-harming behaviors over six months or one year is clinically more relevant in judging g patients’ current safety or risk profile and any improvement. These indicators (e.g., mitigation of recent risk incidents or current safety profile) can be used in determining patients’ readiness for safe transition into the community. To facilitate decision making in forensic psychiatry system, future studies about self-harming behaviors should endeavor to address the prevalence of recent incidents of self-harming behavior and explore the relationship of self-harming behaviour with dynamic and modifiable factors.

The recent study by Jentz et al. [8] may have reported a higher prevalence compared to the current study because it included only females, who have previously been reported to have higher rates of self-harming behaviors [12, 23]. Closely linked is that the forensic system in Canada may be considered among the most developed or better resourced systems globally and more infrastructural safeguards are in place to ensure patients’ safety, thus, the lower prevalence of self-harming behaviors compared to other systems globally. In addition, the forensic system in Canada conducts routine risk assessment with tools such as the electronic-Hamilton Anatomy of Risk Management (eHARM) that may indirectly monitor, and mitigate, self-harming behaviors among patients [24]. The safeguards in use can be implemented in various forensic psychiatric systems globally to ensure safety of forensic patients and detailed descriptions should be provided. In addition, training regarding the implementations of such methods should be initiated for proper and easy adaptation. Despite the known high standards, little descriptions about the safety or risk mitigation protocols, methods, and algorithms used in Canada for patients in the forensic system have been published. Preventive strategies have been proven to be effective in reducing self harming behaviors among individuals in the criminal justice system [25]. We recommend further research to describe such important methods for other areas to learn from. However, in implementation of these methods, it should be noted that the economic and other contextual need of the approaches may be different, and many low-income settings may need context applicable methods. In addition, important lessons and recommendations to prevent self-harming behaviors can be borrowed form other correctional justice system populations [26].

Forensic inpatients in the present study had a higher likelihood of engaging in self-harming behaviors, with inpatients in secure units having higher odds than those in outpatients’ forensic settings. The present findings indirectly underscore the role played by the severity of mental health unwellness and risk of violence. For this reason, individuals who were UST were also at a higher likelihood of self-harming behaviors. Individuals with severe mental health symptoms, especially psychosis, are at a high likelihood of being found UST [27, 28] and may display self-harming behaviors. The trend worsens among individuals with severe forms of neurodevelopmental disorders such as autism, learning disorders, among others; who are involved in self-harming behaviors as a mode of coping with stress or as part of the presentation of their symptomatology [29, 30]. It is also important to note that the forensic psychiatry system has an overrepresentation of individuals with severe psychotic illness, especially schizophrenia as seen in the present study, who may engage in self-harming behaviors due to commands from hallucination or influence of delusions. The presentation of the individuals within the forensic system indicates a high representation of individuals who do not wish to die but are motivated by active symptoms of their illness - a reflection of symptom involvement in some of their index offences.

To the best our knowledge, the present analysis represents the largest study assessing self-harming behaviors among individuals in the forensic systems. Additionally, the dataset was derived from multiple sites and included a wide range of patients and their status (i.e., secure in-patients and outpatients) along the continuum of the forensic system. Despite the size of the database, some study limitations are identified. For example, the present study is retrospective in design and did not specify the type of self-harming behaviors, the methods used, number of incidents, and the severity of the self-harming behaviors and medical outcomes. We recommend future studies to explore such factors or variables to enable the development of a robust strategies to protect the patients better. Second, not all factors related to self-harming behaviors were explored in the present study. Third, this is a retrospective study, and causality can not be inferred. We recommend prospective studies to understand these phenomena. Fourth, the data included was derived from reports covering 2014-15, which may not accurately reflect the current state of self-harming behaviors in the forensic psychiatry system. We recommend the use of recent data in determining the accurate burden of self-harming behaviors. Moreover, future studies using adequately powered models as well as big data analysis to explore the composite relationship of forensic system-related factors with self-harming behaviors are indicated. Lastly, it is also important to note that the prevalence in the current study may be low because it was based on reported incidents by staff or patients that may be biased to including mainly severe incidents and less severe incidents might be ignored.

### Implications of the study findings

The present study has the following implications as per the various stakeholders.

1. Forensic psychiatry patients.

The study findings imply that patients that are unfit to stand trial or inpatients in the forensic system may have a higher risk of self-harming behaviors. Hence, they may benefit from more support and care from the forensic system to mitigate the risk of self-harming behaviour. In addition, those with these identifiable patient-status factors associated with self-harming behaviors may benefit from a closer observation, more screening, monitoring, and individual tailored management strategies, albeit this can sometimes be perceived as stressful and an infringement of their privacy [31].

2) Forensic psychiatry clinicians.

The study findings imply that clinicians need to be more aware of the forensic system-related factors that may influence the risk of self-harming behaviors among their patients. They may need to complete stratified clinical evaluation and analysis to identify individuals with forensic related factors associated with self-harming behaviors in their practice based on evidence-based research and promotion of protective measures to mitigate self-harming behaviors or actions. Such interventions may include monitoring of the patients and completing nuanced evaluation to better understand and develop interventions to relieve all underlying risk factors for self-harm [32]. Clinicians may also need to collaborate with other stakeholders in the forensic system, such as the review board, the legal system, and the correctional system, to ensure the safety and well-being of their patients.

3) Forensic psychiatry researchers.

Based on the study findings, mechanisms and pathways that link the forensic system-related factors and self-harming behaviors among forensic patients need to be explored further. Importantly, there is a need to conduct more studies with larger and more diverse samples using advanced and more rigorous methodology (e.g., use of longitudinal and experimental study designs, to establish causal relationships and test interventions). Large language models may also be used to assist in identifying unit links between the plausible risk factors and self-harm. As research in this area evolves, a detailed exploration of the various forms of self-harm (especially emotional self-harm with behavioural manifestations that could easily be missed) should be explored [33, 34]. This may provide key insight into understanding the warning signs and developing of preventative measures against physical self-harming behaviors. Furthermore, the linkage between the different types or nature of self-harm can be explored to better understand the phenomenon and develop potential interventions. Lastly, dissemination of research findings and recommendations to policy makers, practitioners, and the public is indicated for easy translation.

4) Forensic psychiatry policy makers.

Active engagement of policy makers is needed to promote allocation of adequate resources to provide care for individuals that need indicated intervention or support based on their risk profile for self harm. This is particularly important for patients found unfit to stand trial and inpatients given the present study finding. The resources can support clinical monitoring, staffing, system-related changes and promotion of research targeted mitigating the risk of self-harming behaviors.

5) The general public.

Public health education and support for families or caregivers to understand the need of patients, especially those transitioning into the community might be beneficial. Appropriate access to community based resources, including support lines, counseling teams and emergency care for at-risk individuals are encouraged.

6) The review boards.

In addition to ensuring public safety, the boards should emphasis strategies and promote support for mitigation measures for self-harm for forensic patients, especially those with identifiable risk. This can be done by canvassing for support and mandating provision of services for the rehabilitation and management of patients at risk of self-harm.

## Conclusion

Self-harming behaviors are common among forensic patients, but the prevalence in the present study was found to be lower when compared to previous studies. The severity of mental illness in individuals leading to their status in the forensic system i.e., unfit to stand trial or managed in a high secure unit, was the main factor associated with self-harming behaviors. The findings of this study indicate that forensic system related factors, especially those that pertains to the status of individuals in the forensic system (i.e., unfit to stand trial and being an inpatient) are more associated with individuals who are likely to engage in self-harming behaviors among forensic patients. Constant monitoring of all forensic patients with identifiable risk factors for self-harming behaviours is paramount especially among inpatients and those who are unfit to stand trial. It is also important for all experts working in inpatient forensic settings to be cognizant of both management and assessment of risks for self-harming behaviors.

## Data Availability

The datasets will be made available to appropriate academic parties on request from the corresponding author after approval by GAC.
